# Evaluation of the Insecticidal Potential of *Lysinibacillus fusiformis* Against *Drosophila suzukii* Larvae

**DOI:** 10.3390/insects16111090

**Published:** 2025-10-24

**Authors:** Maristella Mastore, Elisa Broggio, Davide Banfi, Ricardo A. R. Machado, Aashaq Hussain Bhat, Sadreddine Kallel, Marcella Reguzzoni, Silvia Quadroni, Maurizio F. Brivio

**Affiliations:** 1 Laboratory of Applied Entomology and Parasitology, Department of Theoretical and Applied Sciences, University of Insubria, 21100 Varese, Italy; maristella.mastore@uninsubria.it (M.M.); broggio.eli@gmail.com (E.B.); davide.banfi@uninsubria.it (D.B.); 2 Experimental Biology Research Group, Institute of Biology, Faculty of Sciences, University of Neuchâtel, CH-2009 Neuchatel, Switzerland; rarm.machado@gmail.com; 3 Department of Biosciences, University Centre for Research and Development, Chandigarh University, Gharuan 140413, India; aashiqhussainbhat10@gmail.com; 4 Laboratory of Bio-Aggressor and Integrated Protection in Agriculture, Department of Plant Health and Environment, National Agronomic Institute of Tunisia, University of Carthage, Tunis 1054, Tunisia; sadreddine.kallel@inat.ucar.tn; 5 DIMIT, Department of Medicine and Technological Innovation, University of Insubria, 21100 Varese, Italy; marcella.reguzzoni@uninsubria.it; 6 Laboratory of Ecology, Department of Theoretical and Applied Sciences, University of Insubria, 21100 Varese, Italy; silvia.quadroni@uninsubria.it

**Keywords:** spotted-wing *Drosophila suzukii*, biological control, insect midgut, insect immunity, toxins

## Abstract

The increase in the world population and consequent rise in food demand have led to the extensive use of chemical pesticides, causing environmental and health concerns. In response, biological control methods, particularly those involving microbial agents, have emerged as sustainable alternatives within integrated pest management. This study highlights the potential of *Lysinibacillus fusiformis* as a biocontrol agent against the dipteran *Drosophila suzukii* (Matsumura) (Diptera: Drosophilidae), a pest responsible for damaging soft-skinned fruits. Experimental treatments using vegetative cells, spores, and secondary metabolites of *L. fusiformis* on *D. suzukii* larvae demonstrated significant larvicidal effects, accompanied by observable changes in gut morphology under microscopy. Moreover, preliminary immunological assays showed the interference of this bacterium with the host immune system. All the results indicate the suitability of *L. fusiformis* for its possible integration into sustainable agricultural practices, although additional research is required to understand its applicability in the field.

## Simple Summary

The spotted-wing drosophila (*Drosophila suzukii*) is a major pest of thin-skinned fruit crops, causing substantial economic losses and driving increased reliance on chemical pesticides. This study investigated the larvicidal activity of the Gram-positive bacterium *Lysinibacillus fusiformis* against *D. suzukii*. Larvae were exposed to different bacterial components, including vegetative cells, spores, and secondary metabolites. All components exhibited larvicidal effects, with the strongest impact observed when applied in combination. The treatments induced gut damage and impaired immune responses. These findings suggest the potential of *L. fusiformis* as a biological control agent, which may contribute to more eco-sustainable agricultural practices. However, additional research is necessary to evaluate its safety and effectiveness under semi-field and field conditions.

## 1. Introduction

The increasing global demand for food has led to an increased dependence on chemical pesticides, which poses significant risks to the environment, biodiversity, and human health [1,2,3]. To counteract this, sustainable approaches like biological control using living organisms or their metabolites are gaining attention [4,5]. Microbial bioinsecticides (such as bacteria, fungi, and viruses) show strong potential for integrated pest management. However, to use these methods safely and on a large scale, advances are needed in formulation, adaptability, technological integration, and understanding of the interactions between pests or plants and microbes [6,7,8]. Other bacterial biocontrol agents, for example, *Bacillus thuringiensis* (Bt), effectively control pests by competing for nutrients, producing toxins, and boosting plant immunity [7,9]. Bt toxins (Cry and Cyt proteins) specifically target harmful insects and nematodes without affecting non-target species [10,11,12,13]. However, rising pest resistance has prompted efforts to develop new strains, better formulations, and toxin combinations [10,14].

*Lysinibacillus* spp., a Bacillaceae genus, is emerging as a promising alternative to Bt in biocontrol [15]. These Gram-positive, spore-forming bacteria exhibit resilience to environmental stress and function as both bioinsecticides and plant probiotics, since some strains can induce systemic resistance in plants [15,16]. *Lysinibacillus sphaericus* has been commercialized for controlling mosquito vectors [17,18,19]; its larvicidal toxins, such as Bin, Mtx, and Cry48Aa/49Aa, have greater efficacy than those of Bt var. *israelensis* [10,20,21].

Other species, such as *Lysinibacillus fusiformis*, are currently studied for their ability to control Diptera, Coleoptera, and Lepidoptera larvae while improving crop resistance to stress factors [22]. Its probiotic characteristics contribute to plant growth and resilience by improving physiology and systemic resistance to phytopathogens [23,24,25]; the overall properties of *L. fusiformis* make it a suitable candidate both as a biocontrol agent and as a plant growth promoter [26,27,28]. Despite the positive features of this bacterium, the characterization of the biological properties remains incomplete. Further research is needed, starting with controlled laboratory experiments, followed by greenhouse trials and field evaluations.

In this context, the current investigation evaluates a strain of *L. fusiformis*, recently isolated from the soil nematode *Oscheius tipulae* OC2 [29], against the spotted-wing *Drosophila suzukii* (Matsumura) (Diptera: Drosophilidae), a pest responsible for severe damage to soft-skinned fruits [30].

After characterizing the bacterial life cycle, larval survival rates following exposure to vegetative cells, spores, and secondary metabolites of *L. fusiformis*, administered singly or in combination, were assessed. The anatomy of the treated larvae, in particular the morphological alterations in gut structures were examined using bright-field, fluorescence, and scanning electron microscopy (SEM). Furthermore, preliminary immunological analyses were performed to assess hemocyte population dynamics and phagocytic activity in *D. suzukii* larvae following the oral ingestion of the bacterium.

The main objective of this study was thus to investigate the larvicidal and immunomodulatory efficacy of *L. fusiformis* against *D. suzukii* and clarify its potential role in sustainable pest control strategies.

## 2. Materials and Methods

### 2.1. Chemicals and Instruments

All reagents were procured from Sigma Chemicals (St. Louis, MO, USA), and Merck Millipore Ltd. (Tullagreen, Cork, Ireland). Instruments were provided by Bio-Rad Laboratories (Detroit, MI, USA), Euroclone S.p.A. (Milan, Italy, EU), Olympus (Segrate, Italy) and Optika S.r.l. (Ponteranica, Italy). Centrifugations were carried out with a SIGMA 1–14 microcentrifuge (SciQuip Ltd., Newtown, UK) and an Eppendorf 5804 centrifuge (Eppendorf, Hamburg, Germany). Spectrophotometric analysis was performed with a Jasco V-560 spectrophotometer (Jasco, Easton, MD, USA). All materials, buffers, and solutions were either autoclaved or filtered using 0.22 μm Minisart filters (Sartorius, Goettingen, Germany).

### 2.2. Bacteria Isolation

*L. fusiformis* was isolated from the soil nematode strain *Oscheius tipulae* OC2 [29]. To isolate bacteria from nematodes, approximately 200 nematodes were washed several times with sterile PBS (1 mM KH_2_PO_4_, 1 mM K_2_HPO_4_, 5 mM NaCl, pH 7.2) and incubated in a 1% (*v*/*v*) sodium hypochlorite/PBS solution with gentle orbital agitation for 5 min. Nematodes were recovered by centrifugation at 150× *g* for 5 min at room temperature. This procedure was repeated several times to remove contaminants from the nematode epicuticle. Finally, nematodes were washed thoroughly with sterile PBS to eliminate residual hypochlorite. The nematodes were sonicated in sterile PBS at 4 °C for 30 s using a 100 W burst on an ultrasonic processor (Labsonic-L, B-Braun Biotech Inc., Allentown, PA, USA). The fragmented nematodes were collected by centrifugation at 650× *g* for 10 min at 4 °C. A 100 μL aliquot of the supernatant was inoculated into Luria–Bertani (LB) broth and incubated overnight at 30 °C with continuous shaking at 180 rpm. Samples of the culture were then plated onto solid LB agar and incubated at 30 °C for 24 h. Individual colonies were subcultured and used for further experiments.

Bacterial strains were identified as described by Loulou et al. [31], using 16S rRNA gene sequencing and/or whole-genome sequencing by means of the EzBioCloud identification service (https://www.ezbiocloud.net/, 6 March 2022) and the Type (Strain) Genome Server TYGS (https://tygs.dsmz.de/, 6 March 2022) [32,33]. Whole-genome sequences were obtained according to the procedure outlined by Machado et al. [34].

Cytomorphological analyses of *L. fusiformis* were performed on cultures at 1, 2, 3, 4, 6, 24, 30, 48 and 72 h using light microscopy. Bacterial cells were stained using Gram-staining and further stained with malachite green to identify endospores and spores.

### 2.3. Target Insect

*D. suzukii* larvae were obtained from successive generations of a wild population collected in 2012 in Maresme, Catalonia (northeastern Spain). The insects were reared on a standardized diet [35] and maintained in a climatic chamber at 25 ± 1 °C and 45 ± 5% relative humidity (RH), under a 12:12 h light-dark photoperiod. Only healthy larvae at comparable developmental stages (first and second instars, L1 and L2) were selected for the assays. All experimental procedures were carried out under the same environmental conditions.

### 2.4. L. fusiformis Cultures for Bioassays

Cultures of *L. fusiformis* were set up to obtain different mixtures of vegetative cells, spores, and secondary metabolites at varying concentrations to be administered to *D. suzukii* larvae. Bacterial cultures were incubated at 30 °C in the dark with shaking, for durations ranging from 24 h to 7 days. Bacterial growth was monitored by spectrophotometric measurement of biomass at 600 nm (OD_600_).

Post-incubation, vegetative cells (V), spores (S), and secondary metabolites (M) were separated by centrifugation at 950× *g* for 15 min at 20 °C. The presence of bacterial cells and spores was assessed using Gram-staining and light microscopy. Bacterial pellets and supernatants (culture broth) were collected separately. Before use, pellets were washed twice with fresh medium, and bacterial concentrations were determined using the colony forming unit (CFU) method on agar plates via serial dilution and a Cytosmart cell counter (Corning, One Riverfront Plaza, NY, USA).

Supernatants containing secondary metabolites from *L. fusiformis* cultures, harvested after 7 days of culture, were subjected to several centrifugation and filtration cycles through 0.22 µm Minisart filters, to eliminate any remaining contaminants. Subsequently, they were dialyzed against 10 mM Tris-HCl buffer (pH 7.4) and lyophilized. Protein concentrations were determined using the Bio-Rad Protein Assay Kit (Bio Rad cat. 5000006), following the manufacturer’s instructions.

### 2.5. Larvicidal Activity Against D. suzukii

To evaluate the larvicidal potential, early-stage *D. suzukii* larvae (L1–L2) were exposed to vegetative cells, spores, and secondary metabolites of *L. fusiformis* using an agar trap method. Traps consisted of a Petri dish (Ø 6 cm) containing a thin layer of solidified substrate (1% agar, 5% sucrose in sterile tap water) allowing for easy inspection of larval morphology and viability. Ten *D. suzukii* larvae were placed in each trap and incubated at 25 ± 1 °C and 45 ± 5% RH in a climate chamber. 1 mL bacterial suspension was added to each trap, and larvae feeding on agar ingested the bacteria. Susceptibility to *L. fusiformis* was evaluated after single oral administrations of vegetative cells, spores, or secondary metabolites.

First tests were carried out using all bacterial components. Larvae were treated with various concentrations (5 × 10^5^, 1 × 10^6^, 1.5 × 10^6^, and 4 × 10^6^ CFU/mL) of a 24 h culture broth containing vegetative cells, spores, and secondary metabolites.

Further assays were performed without secondary metabolites (vegetative cells and spores only) at the same concentrations (5 × 10^5^, 1 × 10^6^, 1.5 × 10^6^, and 4 × 10^6^ CFU/mL). Additionally, *D. suzukii* larvae were exposed to various concentrations of isolated spores (1 × 10^6^, 1 × 10^7^, and 1 × 10^8^ spores/mL) and secondary metabolites (200 µg/mL and 1 mg/mL) from 7-day bacterial cultures.

In all the assays, concentrations of bacteria and spores were quantified by a cell counter and adjusted to the desired concentrations accordingly. Larvae were incubated in a climate chamber at 25 °C, and survival was recorded at 24, 48, 72 and 144 h post-treatment. As a control, 1 mL of sterile tap water was administered to *D. suzukii* larvae. For each treatment group, 10 larvae, previously surface-sterilized, were used per replicate, and all assays were performed in six independent replicates for each concentration.

### 2.6. Electrophoretic Analysis (SDS-PAGE)

To examine the profile of secondary metabolites from the 7-day cultures of *L. fusiformis*, one-dimensional SDS-PAGE (10%) was carried out with slight modifications of the Laemmli method. Samples were first precipitated with acetone and then resuspended in Laemmli buffer. Aliquots containing 20 µg of protein per well were loaded onto gel slabs using a Bio-Rad Protean IIxi cell (Bio-Rad Laboratories, Hercules, CA, USA). Electrophoresis was carried out overnight at a constant voltage of 30 V. Protein patterns were visualized by silver staining, and molecular weights were determined according to the Weber and Osborn protocol.

### 2.7. Morphology of Treated D. suzukii Larvae

*D. suzukii* larvae, both untreated and treated with *L. fusiformis*, were incubated at 25 °C for 24 h in a climatic-controlled chamber and examined under a stereomicroscope, as well as by light microscopy, fluorescence microscopy, and scanning electron microscopy (SEM).

To preserve gut integrity prior to observation, larvae were euthanized by freezing at −20 °C for 10 min, followed by washing with sterile distilled water. After treatment with *L. fusiformis* spores, the peritrophic membrane expelled from the larvae was collected, stained with Gram-stain and examined microscopically. To evaluate potential tissue damage, gut morphology of *D. suzukii* larvae (L1 stage) was examined following treatment with fluorescent dye TRITC-Dextran (70 kDa). For this assay, 200 µL of a TRITC-Dextran solution (1 mg/mL) was dissolved into the agar-based substrate before solidification.

Larval treatments were performed by supplementing the agar substrate with 200 µL of a suspension containing vegetative cells and spores of *L. fusiformis* (4 × 10^6^ cell/mL), conjugated with fluorescein isothiocyanate (FITC, 1 mg/mL). Larvae were then incubated at 25 °C in a climate-controlled chamber and observed at 1 and 48 h post-treatment. Control larvae were exposed to a substrate containing only TRITC-Dextran. Following treatment, larvae were gently rinsed with physiological buffer, mounted on microscope slides, and covered with a coverslip. Observations were carried out using fluorescence microscopy (Olympus IX-51 microscope) equipped with an OPTIKA C-P20CM digital camera.

Both larvae and isolated gut samples were prepared for morphological observation using SEM. Whole larvae were initially frozen at −20 °C for 5 min prior to preparation. To isolate guts, larvae were dissected and longitudinally opened to expose the mucosal surface, which was then sectioned into smaller fragments. All dissections were performed under aseptic conditions using sterile scissors and forceps. Tissue samples were immersed in 0.9% saline solution and immediately fixed in 1% glutaraldehyde (HiMedia, Thane, India), prepared in 0.1 M sodium phosphate buffer (pH 7.3). Fixation durations were 5 min for intestinal tissues and 1 h for whole larvae, followed by multiple washes with distilled water for 5 min. Samples were post-fixed with 2% osmium tetroxide in collidine buffer for 3 h, then washed with 0.1 M cacodylate buffer. Dehydration was carried out by immersing the samples in a graded ethanol series (70%, 80%, 95%, and 100% absolute ethanol) for 5 min each. For final drying, specimens were treated with hexamethyldisilazane for 5 min, and subsequently air-dried. Specimens were then sputter-coated with gold using an ion-sputtering device and examined using a GeminiSEM 360 scanning electron microscope (Carl Zeiss, Oberkochen, Germany).

### 2.8. L. fusiformis Effect on D. suzukii Hemocytes

Preliminary assays were performed to assess whether *L. fusiformis* could be recognized and neutralized through phagocytosis by *D. suzukii* hemocytes, and to determine whether oral uptake of the bacteria affected total hemocyte count. As described in Section 2.5, larvae were treated with FITC-labeled *L. fusiformis* spores at a concentration of 1 × 10^8^ spores/mL. Control larvae were fed a substrate supplemented with TRITC-Dextran (1 µg/mL) to assess its uptake and establish a temporal baseline for bacterial ingestion. All larvae were incubated at 25 °C in a climate-controlled chamber under dark conditions. To evaluate phagocytic activity, hemolymph was collected from 60 larvae (L1/L2 instar), 4 and 12 h post-bacterial ingestion. Larvae were washed with sterile PBS, surface-sterilized with 70% ethanol, anesthetized at 4 °C, and dorsally dissected using micro-dissection scissors. Dissected larvae were placed in PCR tubes perforated at the bottom with a needle and inserted into 500 µL Eppendorf tubes for hemolymph collection via centrifugation. To inhibit phenoloxidase activation, 10 µL of saturated phenylthiourea (PTU) solution was added to each hemolymph sample. Samples were centrifuged at 180× *g* for 5 min at 4 °C. The supernatant was discarded, and the hemocyte pellet was resuspended in Schneider medium. Hemocytes were washed twice to remove tissue debris and bacterial contaminants and then plated in 96-well MicroWell™ plates (Thermo Fisher Scientific, Waltham, MA, USA) at a final concentration of 2 × 10^5^ cells/mL. Cells were incubated for 30 min at 25 °C in the dark, nuclei were stained with DAPI and subsequently observed under a fluorescence microscope to assess phagocytic activity.

For the quantification of total hemocytes, hemolymph was extracted from 10 larvae and placed onto a glass slide containing 100 µL of Schneider’s medium supplemented with 10% saturated PTU solution. Ten microliters of the mixture were loaded onto a Neubauer hemocytometer, and hemocytes were counted at 5× magnification using a Corning cell counter (Corning Inc., New York, NY, USA). Data were processed using Axion BioSystems AxIS Vue version 33.12 software. Counts were performed at 4 and 12 h post-ingestion, using untreated larvae as controls. All assays were repeated five times.

### 2.9. Data Analyses

Survival analyses for each treatment group of *D. suzukii* larvae, infected with different concentrations of *L. fusiformis*, were conducted using the Kaplan–Meier estimator. Overall differences in survival patterns between concentrations were evaluated using the log-rank (Mantel–Cox) test. Test statistics were evaluated against a chi-square distribution to determine statistical significance (α = 0.05), using GraphPad Prism 8.4.2. Subsequently, the difference in mean mortality of *D. suzukii* larvae exposed for 72 h (i.e., evaluation of the short-term effect) to the highest tested concentration of each treatment was statistically assessed using one-way analysis of variance (ANOVA), followed by Tukey’s post hoc test for pairwise comparisons, in PAST 5.2. These statistical tests were also used to assess possible significant differences in hemocyte counts between control larvae and those infected with *L. fusiformis* spores after 4 and 12 h post-treatment.

## 3. Results

### 3.1. L. fusiformis Isolation and Culture

**Figure 1:**
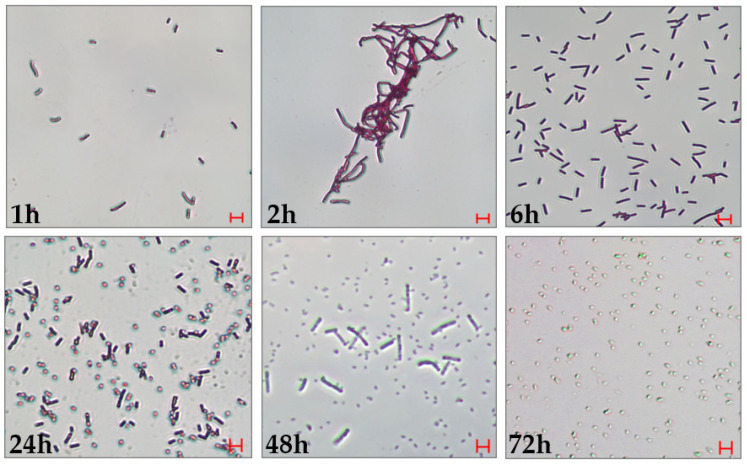
*L. fusiformis* life cycle in culture. The growing bacterial culture was monitored at various times between 1 and 72 h. It is possible to observe the presence of vegetative cells up to 6 h after inoculation, while in the interval 24–72 h spores can be detected, becoming prevalent at longer times (72 h of culture). Bar = 5 mm.

To characterize the developmental cycle of *L. fusiformis*, in vitro cultures were monitored under a light microscope following Gram-staining at times ranging from 1 to 72 h post-inoculation (Figure 1). At 1 h, single vegetative rod-shaped cells were observed. Between 2 and 6 h, active proliferation and elongation of vegetative cells were evident. From 24 to 48 h, the culture revealed sequential stages of germination, outgrowth, and spore formation. At 72 h, cultures were composed exclusively of spores, indicating the achievement of the sporulation process.

### 3.2. Effects of L. fusiformis Administration to D. suzukii Larvae

**Figure 2:**
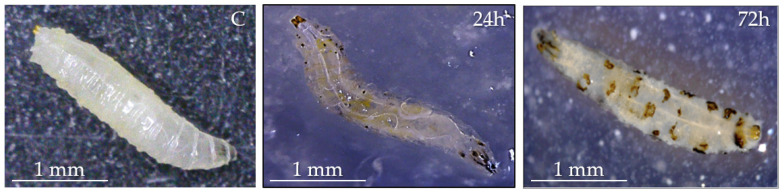
*D. suzukii* infected with *L. fusiformis* (vegetative cells, spores, secondary metabolites, 24 h of culture), 24 and 72 h after treatment. Morphological changes are observed in the post-infected larvae; spots of melanization are evident 72 h post-infection. C: control larvae. Bar = 1 mm.

A preliminary test was carried out to evaluate the intake and pathogenic properties of *L. fusiformis*. Figure 2 shows the morphology of infected larvae at 24 and 72 h post-treatment. After 24 h, the larvae exhibited initial signs of melanization, appearing as dark spots on the body. By 72 h, their health was visibly affected, with widespread melanization and a marked decrease in motility.

**Figure 3:**
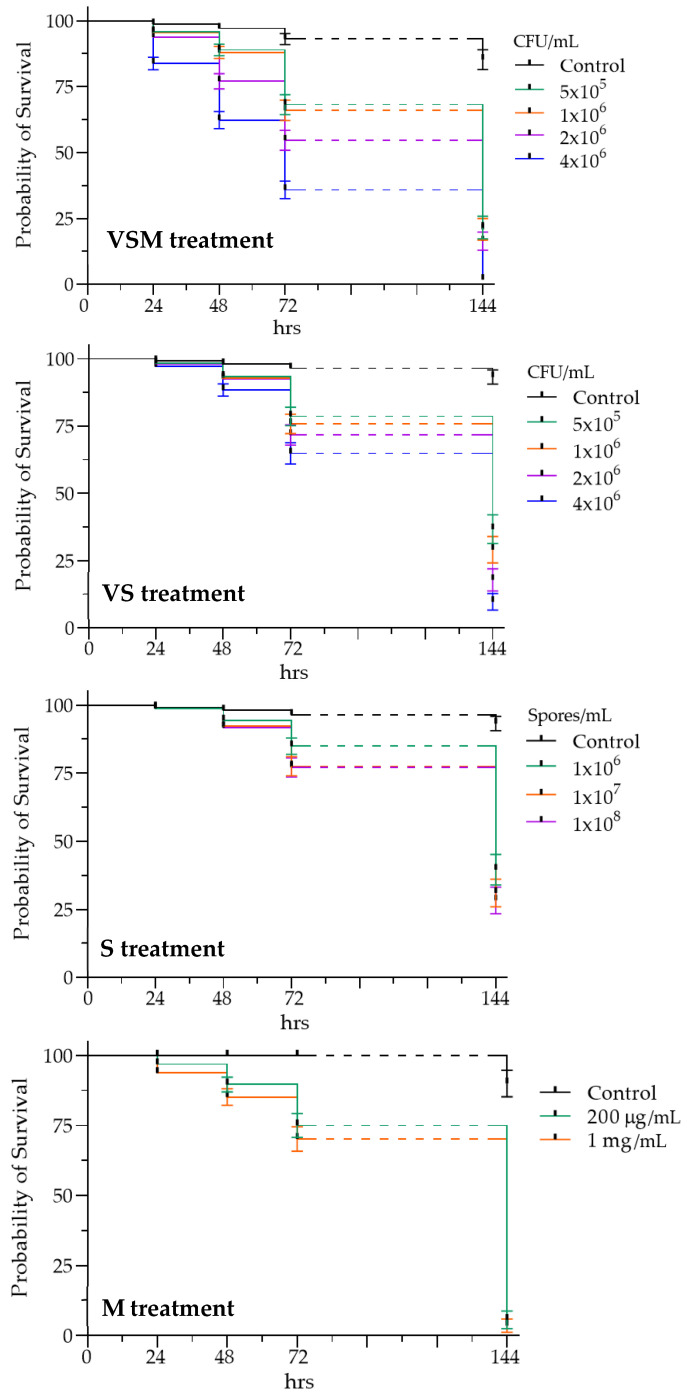
Kaplan–Meier survival curves of *D. suzukii* larvae after different treatments at different concentrations: with vegetative cells, spores and secondary metabolites from cultures of *L. fusiformis* (from 5 × 10^5^ to 4 × 10^6^ CFU/mL, VSM), with vegetative cells and spores (from 5 × 10^5^ to 4 × 10^6^ CFU/mL, VS), with spores alone (10^6^, 10^7^ and 10^8^ spores/mL, S) and with secondary metabolites alone (200 µg/mL and 1 mg/mL, M). Survival rate decreases with increasing bioinsecticide concentration in each treatment. Dotted line refers to the time interval in which mortality was not monitored. A significant difference between the survival curves corresponding to the different tested concentrations was detected for each treatment (log-rank test, VSM: χ^2^ = 231.6, df = 4, *p* < 0.0001; VS: χ^2^ = 120.8, df = 4, *p* < 0.0001; S: χ^2^ = 72.08, df = 3, *p* < 0.0001; M: χ^2^ = 99.57, df = 2, *p* < 0.0001).

The effects of each treatment were monitored at 24, 48, 72, and 144 h and are reported as Kaplan–Meier survival curves (Figure 3).

Larvae treated with the complete bacterial mixture, including vegetative cells, spores, and secondary metabolites (i.e., VSM treatment), showed a relatively high probability of survival at the lower tested concentrations (5 × 10^5^ and 1 × 10^6^ CFU/mL) until 72 h, with survival falling below 50% only at 144 h. A more rapid decline in survival probability was observed at the highest tested concentration (4 × 10^6^ CFU/mL), falling below 50% at 72 h.

**Figure 4:**
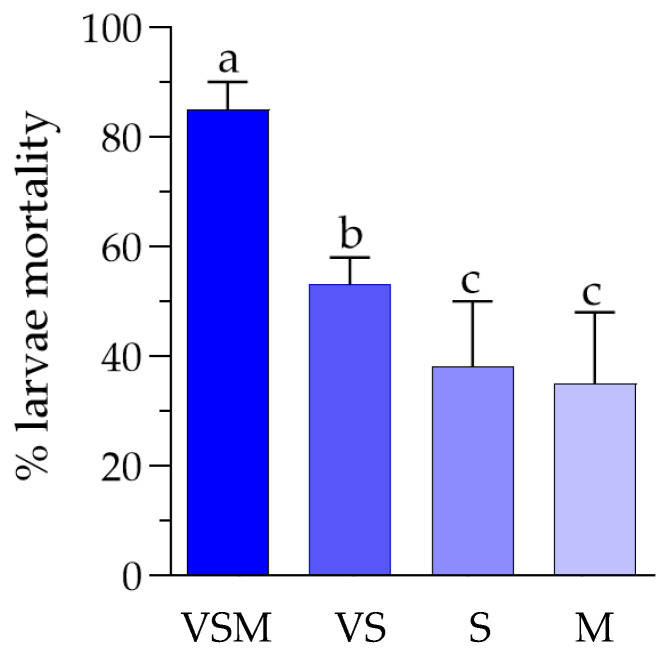
Comparison between the mortality of *D. suzukii* larvae exposed for 72 h to different treatments: VSM = complete bacterial mixture including vegetative cells, spores, and secondary metabolites at 4 × 10^6^ CFU/mL; VS = bacterial mixture including vegetative cells and spores at 4 × 10^6^ CFU/mL; S = spores at 1 × 10^8^ spores/mL; M = secondary metabolites at 1 mg/mL. An overall significant difference between treatments was detected (one-way ANOVA test: F = 35.5, *p* < 0.0001). Moreover, different letters indicate significant pairwise differences assessed using Tukey test (*p* < 0.05).

The evaluation of *D. suzukii* larval survival following exposure to vegetative cells and spores (i.e., VS treatment) showed that the absence of secondary metabolites led to reduced insecticidal activity, with survival remaining above 50% at all tested concentrations even after 72 h. Similarly, treatments with spores alone (i.e., S treatment) or secondary metabolites alone (i.e., M treatment) demonstrated a significant efficacy only at 144 h. A direct comparison of *D. suzukii* larval mortality after 72 h of exposure (short-term effect) to different treatments (only the highest concentration for each treatment was selected for the comparison) confirmed the significantly higher lethal effect of the complete bacterial mixture, with average mortality of 85%, followed by the combination of vegetative cells and spores (average mortality of 53%). Treatments with either spores or secondary metabolites alone were less effective, showing average mortality rates of 38% and 35%, respectively (Figure 4).

**Figure 5:**
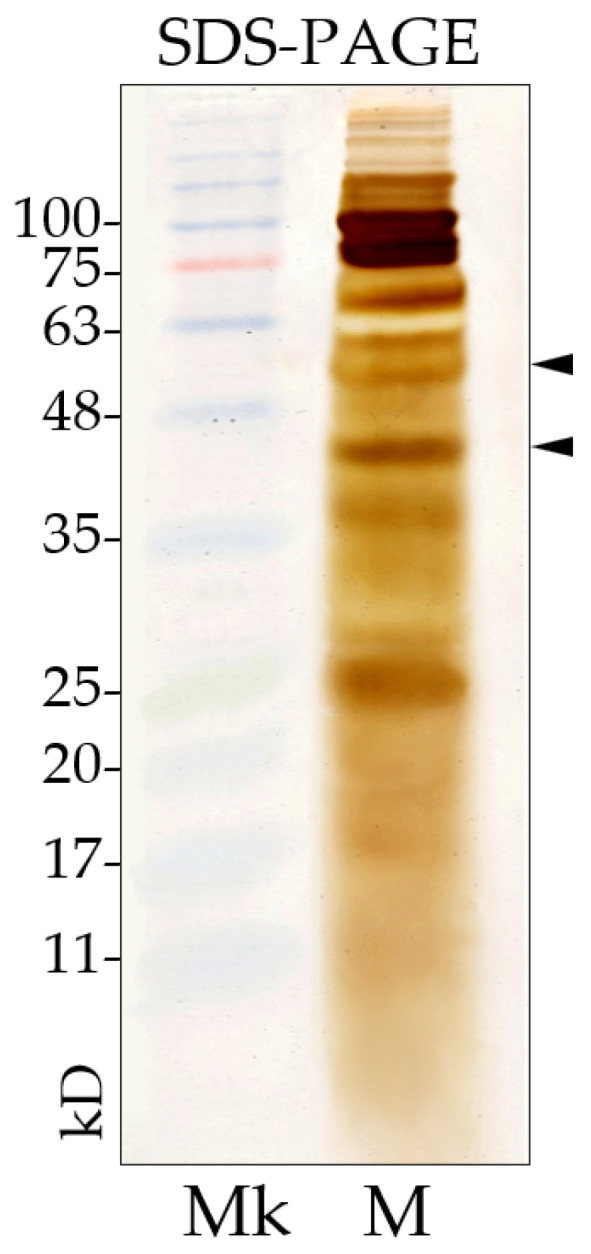
SDS-PAGE (10%) of *L. fusiformis* metabolites from 72 h culture. Analytical electrophoresis shows several bands, two of which, at approximately 42 kDa and 52 kDa (arrowheads). Mk = Marker; M = secondary metabolites.

The protein profile of *L. fusiformis* secondary metabolites (Figure 5), obtained from a 72 h culture, was analyzed using SDS-PAGE. Analytical electrophoresis revealed the presence of multiple bands, including two with apparent molecular masses of 42 kDa and 52 kDa.

### 3.3. Effects of L. fusiformis on the Gut of D. suzukii Larvae

**Figure 6:**
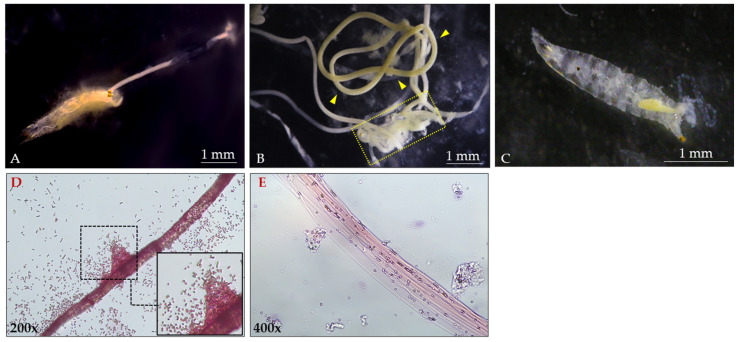
*D. suzukii* larvae after treatment with *L. fusiformis* spores (10^8^ spores/mL). (**A**): Expulsion of the peritrophic membrane from the anal pad of the larvae, 12 h post administration. (**B**): Peritrophic membrane (arrowheads) completely outspread from the larval body (dashed box) 48–72 h post-treatment. (**C**): Altered anatomy of the larvae 72 h post-treatment, due to the complete loss of the peritrophic membrane. (**D**): Gram-stained peritrophic membrane ejected from the larvae 24 h post administration. (**E**): Acellular structure of the peritrophic membrane after removal of the bacterial mass.

During survival assays with *L. fusiformis* at a concentration of 10^8^ spores/mL, significant alterations in the larval gastrointestinal system were observed. As shown in Figure 6, a tubular structure was extruded from the caudal region. Microscopic analysis identified this structure as the peritrophic membrane, which was expelled between 24 and 48 h post-exposure (Figure 6A,B, indicated by arrows). By 72 h, the larval bodies appeared translucent, with visibly depleted portions of the gastrointestinal tract (Figure 6C).

Figure 6D,E also shows a portion of the isolated peritrophic membrane. Gram-staining revealed a high bacterial load associated with the membrane (panel D and inset). In panel E, following bacterial removal, the acellular nature of the peritrophic membrane is clearly visible.

**Figure 7:**
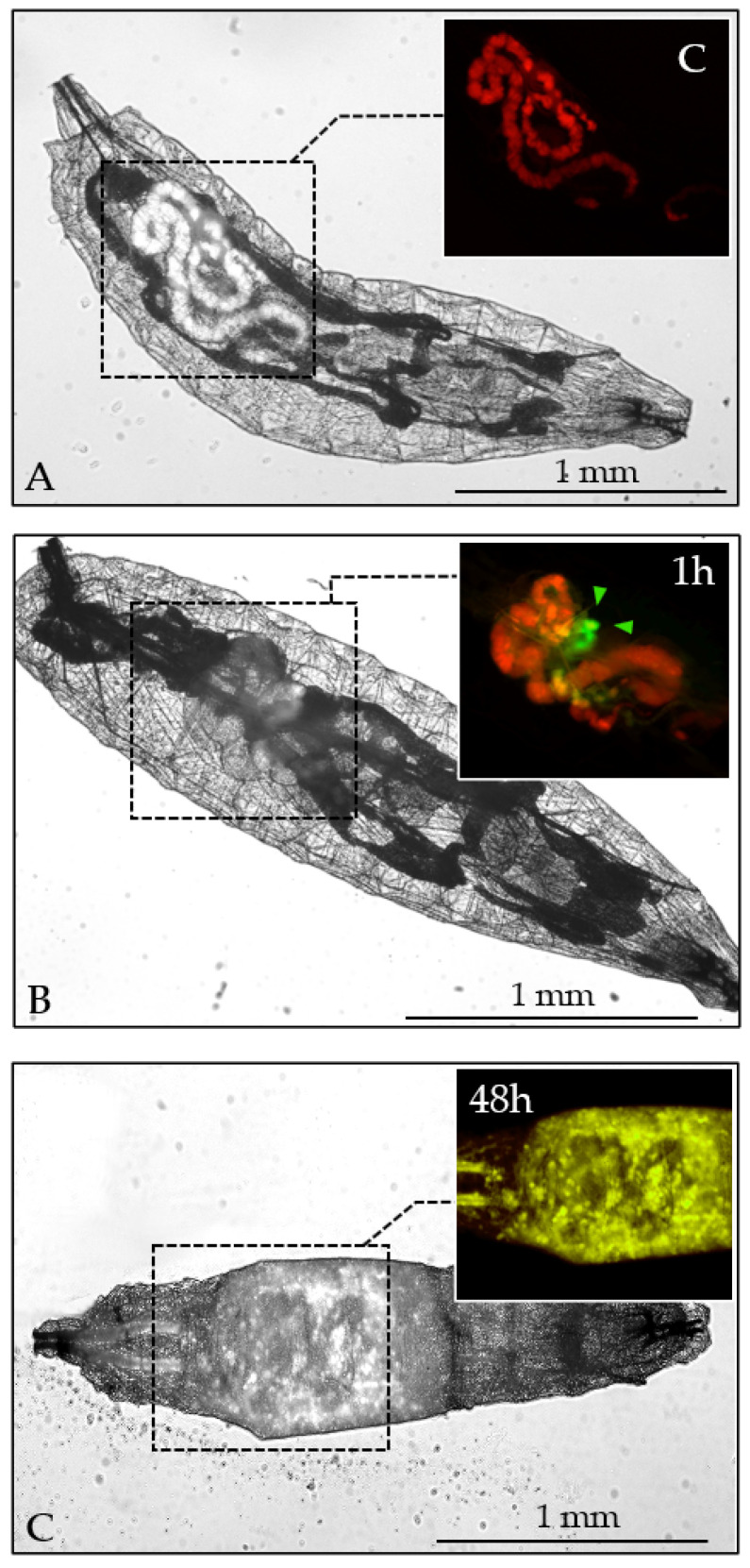
Fluorescence microscopy of larvae. (**A**): Control larvae (Dextran-TRITC, 24 h) display normal gut anatomy. (**B**): Treated larvae (Dextran-TRITC and *L. fusiformis*-FITC, 1 h) show no evident gut damage. The inset highlights the presence of ingested Dextran-TRITC (red) and the localization of *L. fusiformis* (green arrows) within the midgut. (**C**): At 48 h post-infection, larvae exhibit structural damage to the gut, with bacterial fluorescence extending beyond the gastrointestinal tract (inset). Bar = 1 mm.

Further investigations using fluorescence microscopy were carried out to assess the effects on the gut of *D. suzukii* larvae following oral ingestion of bacteria (Figure 7).

Panel A of Figure 7 shows a control larva, where ingestion of the substrate agar-dextran-TRITC is localized in the gut lumen, which appears unaltered (inset). One hour after the addition of FITC-labelled *L. fusiformis* to the substrate (Figure 7, panel B), the presence of bacteria was evident in an area of the midgut (inset, green arrows), and dextran-TRITC fluorescence confirmed gut integrity. Larval anatomy was monitored up to 48 h (Figure 7, panel C). At this time, widespread bacterial distribution was observed throughout the central region of the larval body. The presence of both bacterial and dextran fluorescence outside the gastrointestinal tract (inset) suggested probable gut damage. The observed yellow-green fluorescence represents a merged signal from both dextran and bacterial emissions.

### 3.4. Scanning Electron Microscopy of Control and L. fusiformis-Treated D. suzukii Larvae

**Figure 8:**
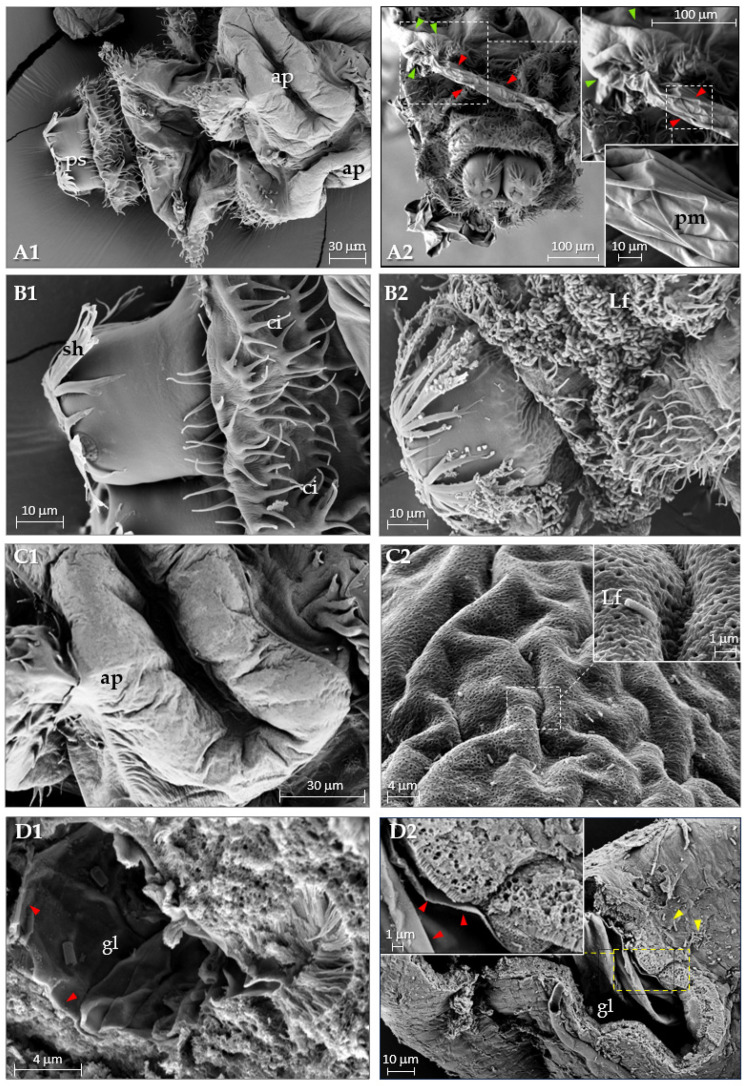
Scanning electron microscopy (SEM) of untreated (**A1**,**B1**,**C1**,**D1**) and treated (**A2**,**B2**,**C2**,**D2**) *D. suzukii* larvae. (**A1**): Posterior region. (**A2**): The peritrophic membrane (red arrows) protrudes from the anal region (green arrows); inset shows an enlargement of the anal pad, and a further enlargement shows the structure of the peritrophic membrane. (**B1**): Abdominal spiracles in the caudal region. (**B2**): Large numbers of bacteria are observed in the region of the spiracles. (**C1**): Enlargement of the anal pad region. (**C2**): Anal pad; the enlargement (inset) of the anal pad region shows *L. fusiformis* cells. (**D1**): Transverse section of the gut, the peritrophic membrane (red arrows) attached to the intestinal epithelium is observed. (**D2**): In treated larvae, the detachment of the peritrophic membrane into the intestinal lumen is evident. Higher magnification insets illustrate the detachment of the peritrophic membrane (red arrows), while yellow arrows point to the presence of *L. fusiformis* cells. ap: anal pad; ps: posterior spiracles; sh: spiracle hairs; cr: cirri; Lf: *L. fusiformis*; pm: peritrophic membrane; gl: gut lumen.

Using SEM, we analyzed in detail the morphology of the larval caudal region after treatment (Figure 8). The micrographs on the left panels (Figure 8A1,B1,C1) show the caudal region of control larvae, while the corresponding right panels (Figure 8A2,B2,C2) display larvae following treatment. In panels A1, B1, and C1, various external anatomical structures of the larva are visible, including anal pads (ap), posterior spiracles (ps), spiracle hairs (sh), and cirri (ci). Panel A2 shows the posterior region of a larva treated with *L. fusiformis* (Lf) 24 h post treatment. Extrusion of the peritrophic membrane (red arrows) through the anal pad (green arrows) is evident, with insets providing a magnified view of the anal pad region and the extruded peritrophic membrane (pm). The presence of bacteria on the surface of the caudal region of the larva is shown in panel B2. Panel C2 and its inset show a higher magnification of the anal pad colonized by *L. fusiformis*. A transverse section of the midgut from a larva treated with *L. fusiformis* (panel D2, yellow arrows) highlights the detachment of the peritrophic membrane (red arrows) from the gut epithelium. In contrast, in control larvae (panel D1),the peritrophic membrane remains adherent to the gut lumen (gl).

### 3.5. Total Hemocytes Count (THC) and Phagocytosis Assay of L. fusiformis

**Figure 9:**
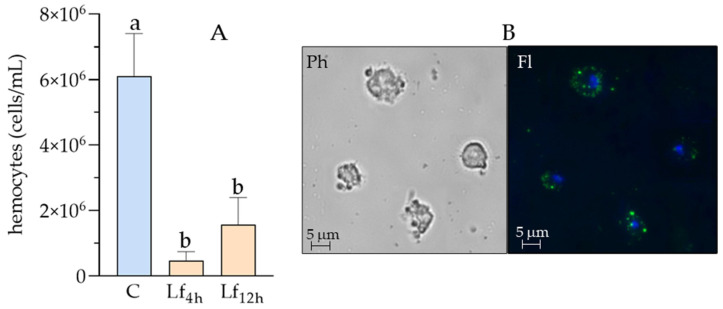
(**A**): total hemocytes count (THC) in *D. suzukii* hemolymph after *L. fusiformis* treatment. C = control; Lf_4h_ = THC 4 h post treatment; Lf_12h_ = THC 12 h post treatment. An overall significant difference between treatments was detected (one-way ANOVA test: F = 33.1, *p* = 0.0006). Moreover, different letters indicate significant pairwise differences assessed using Tukey test (*p* < 0.05). (**B**): in vivo hemocytes phagocytosis of *L. fusiformis* 4 h post treatment. Hemocytes in phase contrast (Ph) show cell damage after bacteria engulfment. Fluorescence image (Fl) shows the presence of FITC-conjugated bacteria inside cells (green spots), and nuclei (blue spots).

The hemocytes population of *D. suzukii* was examined in both control larvae and those treated with *L. fusiformis* spores. Changes in total cell count are illustrated in Figure 9A. A significant decrease was recorded at both 4 h (4.67 × 10^5^ cells/mL) and 12 h (1.6 × 10^6^ cells/mL) after infection, compared to the control (6.1 × 10^6^ cells/mL).

To evaluate the phagocytic activity of *D. suzukii* hemocytes (Figure 9B), hemolymph was extracted 4 h after oral administration of *L. fusiformis*. Hemocytes were separated from the humoral fraction, and the results showed their phagocytic capability (Fl) after engulfment of FITC-conjugated bacteria. Cell damage caused by the presence of bacteria was evident under phase-contrast microscopy (Ph).

## 4. Discussion

Various multifunctional organisms, particularly antagonistic ones, offer promising solutions for controlling harmful insects, improving plant growth, maintaining ecological balance, preventing resistance, and reducing risks to human and other animal health [36,37,38]. Microorganisms commonly used in biological control often play multiple roles including pathogen control, biostimulation and biofertilization [39,40,41]. These bacterial bioproducts come in various formulations, such as single cells or spores, and may contain one or more species in viable or bioactive forms. When applied to plants or soil, they improve crop physiology and help control pests and pathogens [42,43]. Rhizobacteria are of growing interest [44], including certain species of *Lysinibacillus*, which have several positive features such as entomopathogenicity, disease control, plant growth promotion, and bioremediation [45,46].

In this study, we evaluated the potential of *L. fusiformis*, a scarcely investigated species, as a biocontrol agent against the invasive fruit fly *D. suzukii*, specifically assessing its effects on larval survival, gut integrity, and immune response. The tests were carried out on L1 and L2 larvae, as these early developmental stages exhibit high feeding activity and increased susceptibility to infection due to their immature immune systems. *D. suzukii* larvae develop within the fruit, making it challenging for bioinsecticides to reach them effectively. However, egg hatching and the earliest larval stages occur near the fruit surface, close to the lesion created by the female ovipositor; this initial positioning makes the early stages more vulnerable than the later stages, which burrow deeper into the fruit pulp as they feed.

Observations on bacterial life-cycle revealed a sequential growth of *L. fusiformis* from vegetative cells to spores within 72 h. This suggests that the bacterium follows a regulated development cycle in vitro, and these observations could be useful for optimizing its future use in greenhouses and fields. Rapid proliferation and sporulation could be crucial for its persistence and spread in the host when administered to target insects. After administration of the bacteria, *D. suzukii* larvae showed early signs of melanization, indicating the triggering of the effector mechanisms of the host immune response against pathogens. After 24 h, the larvae immune response failed to control bacterial proliferation, resulting in the evolution of sepsis. Then, a progressive decrease in motility and an increase in melanized spots on the host body were observed, particularly at the highest bacterial concentrations. Mortality recorded at 72 and 144 h supports the conclusion that *L. fusiformis* possesses entomopathogenic potential.

Survival assay results indicated that the complete bacterial mixture (VSM), including vegetative cells, spores, and secondary metabolites, exerted the most significant impact on larval survival, with a concentration and time-dependent mortality. In this case, mortality rates reached 85% on average at 72 h at the administered concentration of 4 × 10^6^ CFU/mL. Larvae exposed solely to vegetative cells and spores (VS) exhibited lower mortality rates, suggesting that secondary metabolites may play a key role in pathogenicity.

The supernatant of *L. fusiformis* culture was analyzed for secreted proteins using SDS-PAGE. The electrophoretic pattern showed several main protein bands with molecular weights ranging from 15 to 120 kDa. Two of these bands corresponded in size to the BinA and BinB toxins previously identified in *L. sphaericus* which, as described by Lekakaern et al., showed toxicity towards *Culex quinquefasciatus* larvae [47]. Both proteic and non-proteic secondary metabolites may play a role in the bacterium insecticidal activity.

Dahmana et al. evaluated the effects of *L. fusiformis* on *Aedes albopictus* mosquito larvae, by analyzing the metabolites and the cellular fraction from a bacterial culture; the cellular fraction was found to be inactive, while the metabolites caused 85% mortality [48]. Also, Aneha et al. demonstrated the insecticidal activity of *L. fusiformis* against *C. quinquefasciatus* and *Aedes aegipti* [49]. As reported for Bt and *L. sphaericus*, toxins damage the intestinal epithelium [20,50,51,52,53], and *L. fusiformis* seems to exhibit a similar mechanism. However, the effectiveness of *L. fusiformis* does not seem to be based only on the effects of toxins, but also on the contribution of vegetative cells and spores, suggesting a combined action of all the components of the bacterial life cycle.

The peritrophic membrane is a thin protective structure composed of chitin, proteins, and glycosaminoglycans, which lines the midgut of most insects. It acts as a barrier, playing a crucial role in protecting the epithelium from abrasions, pathogens, and toxins [54,55]. Alteration and disruption of this membrane is a common strategy used by many entomopathogenic bacteria, e.g., Bt toxins damage this membrane facilitating pathogen access to the intestinal epithelium, and thus promoting systemic infections [55,56]. The results of this study show that 24 h after administration of *L. fusiformis*, the peritrophic membrane of *D. suzukii* is extruded from the larva. This process can be considered a key event that increases the susceptibility of the intestinal epithelium to the action of the pathogen. Specifically, scanning electron microscopy (SEM) revealed drastic morphological changes in the caudal region of the treated larvae, i.e., detachment from the epithelium and extrusion of the peritrophic membrane, as well as bacterial colonization of the larval body. 48 h after treatment, the intestinal damage becomes more severe. Fluorescence microscopy showed a widespread distribution of bacteria, both in the intestine and in the hemocoel cavity of the larva, suggesting that *L. fusiformis* breaks through the peritrophic membrane barrier and invades the host tissues. Bacterial infiltration and degradation of the intestinal structure contribute to the high mortality observed in treated hosts. As detected in other *Lysinibacillus* species, *L. fusiformis* can secrete secondary metabolites that synergistically contribute to compromising intestinal functions, thus increasing its pathogenicity. This is consistent with the results obtained with *Pseudomonas entomophila*, which secretes virulence factors such as phospholipases and proteases that compromise intestinal integrity [57].

This study also evaluated some processes of the immune response of *D. suzukii* larvae following exposure to *L. fusiformis*. A decrease in total hemocyte count (THC) 4 h after infection suggests that bacteria may compromise the effectiveness of the immune system. A slight but not significant increase in THC 12 h after oral ingestion may indicate an attempt by the larvae to reorganize the immune response by differentiating new immunocompetent cells. Phagocytosis assay results further support the interference hypothesis: while hemocytes do engulf bacteria, notable cellular damage occurs, indicating that bacterial interference with immune functions may be due to their secreted secondary metabolites and enzymes.

A change in the number of hemocytes after exposure to bacteria is a typical consequence of the presence of various pathogens used in pest control; this suggests a general weakening of the host defenses during prolonged infection [58]. Genetic studies on *L. fusiformis* confirmed the presence of virulence-associated genes, including hlyIII and sph, which encode hemolysin III and sphingomyelinase, respectively, both of which participate in cytotoxicity and tissue damage [59]. Similar effects have been observed in *Spodoptera littoralis* infected with *Pseudomonas* species, resulting in hemocyte death and damage to intestinal tissue [60], and in *Galleria mellonella* infected with Bt, which caused a reduction in hemocyte numbers and phagocytic activity [61]. In *L. fusiformis*, secondary metabolites, which may include BinA and BinB toxins, probably contribute to immunosuppression; these molecules can directly compromise hemocyte function or interfere with key signaling pathways required for the activation of further immune processes.

*L. fusiformis* seems to exploit various virulence factors, such as toxins and secondary metabolites to weaken the host immune system, damage gut tissues, and kill the insect host; the synergistic action of its cells, spores, and metabolites may also improve its effectiveness as a biological control agent.

## 5. Conclusions

Although obtained under laboratory conditions, these results contribute to understanding bacterial-insect interactions and support the development of sustainable pest management strategies. Future research should focus on elucidating the molecular mechanisms of host-bacteria interactions, particularly the role of secondary metabolites in pathogenicity. Optimizing bacterial formulations and application methods will be crucial to enhance field performance and prevent resistance in target pests. Additionally, assessing the ecological safety and effects on non-target organisms of *L. fusiformis*, along with long-term field trials and genomic studies, will help realize its full potential in sustainable agriculture.

## Data Availability

The original contributions presented in this study are included in the article. Further inquiries can be directed to the corresponding author.
